# Contemporary eDNA methods complement conventional microscopy in zooplankton diet studies: Case study with American lobster postlarvae

**DOI:** 10.1371/journal.pone.0325889

**Published:** 2025-06-25

**Authors:** Alexander Ascher, Peter D. Countway, Robin S. Sleith, Curtis Morris, Caitlin Haley, David M. Fields, Richard A. Wahle

**Affiliations:** 1 School of Marine Sciences, University of Maine, Orono, Maine, United States of America; 2 Bigelow Laboratory for Ocean Sciences, East Boothbay, Maine, United States of America; Central University of South Bihar, INDIA

## Abstract

The diets of pelagic marine larvae are difficult to analyze due to their small size and even smaller prey. Furthermore, different methods may lead to alternative interpretations of trophic interactions. Conventionally, diet studies have relied primarily on visual identification of prey through dissection and microscopy. While microscopy has clear benefits, it can yield an incomplete assessment of diet since smaller and soft-bodied prey items are often difficult to identify. Here, we combined conventional microscopy and two contemporary environmental DNA (eDNA) methods: DNA metabarcode sequencing (metabarcoding) and real-time Polymerase Chain Reaction (rtPCR), comparing their advantages/disadvantages in a diet analysis of planktonic American lobster *(Homarus americanus)* postlarvae. This is the first application of these molecular techniques on the postlarval lobster diet. We also describe the testing and development of a novel blocking primer designed to inhibit the amplification of lobster DNA, enhancing prey amplification. This approach allowed finer-scale identification of a greater variety of prey than microscopy. The targeted rtPCR approach identified a specific prey taxon with high fidelity – but involves *a priori* decisions regarding the choice of target. Here, an rtPCR assay was developed to target *Calanus finmarchicus,* an abundant copepod species in the Gulf of Maine, suspected to be an important prey item of larval lobsters. Microscopy revealed broad prey categories and the importance of arthropod prey in the postlarval diet. Metabarcoding confirmed the importance of arthropod prey, while filling in the gaps with additional prey species. Finally, rtPCR was able to detect a significant level of predation on *Calanus finmarchicus* that neither of the other two approaches identified. The combination of methods provided a richer understanding of diet than any single method alone and future diet studies of a wide range of consumers would benefit from the application of a mixture of microscopy and molecular-based methodologies.

## Introduction

Knowledge of a predator’s diet is fundamental to the understanding of its ecology [1–3]. Interpreting diet composition through the analysis of stomach contents can reveal important insights into the trophic interactions and energy requirements of species in their natural settings. However, conventional visual analysis of diet can prove difficult as foods are degraded through digestive processes and as the size of the prey decreases [4]. Molecular approaches are becoming increasingly prevalent in the study of consumer food habits in order to overcome these difficulties [2,4,5].

Many benthic organisms like the American lobster (*Homarus americanus*) have larval stages that spend the first several phases of their life in the pelagic zone as meroplankton. The planktonic prey of these organisms exist within complex food-webs that are being altered by a changing climate. Therefore, to understand the ecology of benthic marine species that have pelagic stages, it is critical to capture as complete a picture of their diets as possible during these vulnerable periods of their lives. Consideration of the best approaches for diet analyses has been the subject of numerous studies (e.g., [2,4,6,7]). Novel molecular techniques using environmental DNA (eDNA) approaches have become more common to surmount the challenges of diet analyses (e.g., copepods: [8,9]; spiny lobsters: [10]; spiders: [11]; fishes: [12]; turtles: [13]; mussels: [14]), but these newer methods carry their own sets of limitations and potential biases.

In this paper, we compare the utility of conventional microscopy to two molecular techniques for diet analysis of planktonic organisms. We employed the postlarva of the American lobster (*Homarus americanus*) for this case study, an ecologically and economically important benthic consumer in the northeast US and Atlantic Canada. These methods included visual identification of the gut content of dissected animals by microscopy, as well as two DNA-based approaches: metabarcoding and real-time Polymerase Chain Reaction (rtPCR). This represents the first application of either of these DNA-based approaches towards understanding the postlarval lobster diet.

The American lobster was selected both for its commercial importance and because prior evidence suggests planktonic survival and benthic recruitment may be limited by the planktonic food supply. Specifically, declining benthic recruitment of lobster to coastal nurseries has been linked to a decrease in the abundance of a foundational copepod species in the Gulf of Maine, *Calanus finmarchicus,* suggesting that changes in its availability may be driving the decline [15–17]. Furthermore, benthic recruitment of young-of-year lobster is a predictor of subsequent recruitment to the fishery some 5–8 years later [18–20]. Therefore, to discern whether *C. finmarchicus* may be an important diet constituent of postlarval lobster, we tailored a molecular assay to detect its DNA in postlarval lobster gut samples. Here, we employ postlarval American lobster as a case study in order to introduce and highlight the uses and shortcomings of different diet analysis techniques. The lessons learned may be generally applicable to disentangle pelagic food-webs of other small, planktonic feeders.

### Microscopy

Gut dissection and subsequent visual identification with the unaided eye or through microscopy is the most well-established and conventional methodology in diet studies where direct observation of feeding is not possible or practical [21,22]. Microscopy and visual identification of gut contents has been in broad use for decades and continues to be an important tool for studying trophic ecology and food habits of predators, generating large databases of dietary data [23–25]. Data collected by microscopy can be recorded as presence/absence, total numbers of individuals for each prey species, or the amount of food in the gut measured volumetrically or gravimetrically [2,22]. The level of difficulty associated with gut content identification varies with the size of the study organism and its feeding ecology. For example, the diet of a large fish that captures its prey whole are much easier to discern than the diet of a small crustacean which tears its prey into smaller pieces. Additionally, prey that leave behind hard parts (e.g., bones, exoskeleton) are easier to identify than prey which are primarily soft-bodied.

### DNA tools

Popularity of molecular approaches has increased in contemporary diet analyses as the technology has developed and become more widely available for ecological research [26]. In this study, we used two specific eDNA techniques including DNA metabarcoding (also known as ‘amplicon sequencing’), and real-time Polymerase Chain Reaction (rtPCR).

DNA metabarcoding can be used to identify the genetic diversity within a sample for a selected gene or set of genes [2,27]. It makes use of universal primers that bind to highly conserved DNA regions flanking more variable regions; the variable region then acts as a ‘barcode’ allowing for identification of the genetic diversity within the sample. However, the vast majority of genetic material in a predator’s gut belongs to the predator itself, and so it is imperative that the amplification of this ‘host’ DNA be delayed or suppressed. Failure to do so results in DNA from the predator being preferentially amplified during the PCR [28], causing the less abundant prey DNA to remain unidentified [6,29]. To minimize amplification of the predator DNA, a predator-specific blocking primer can be employed to reduce its amplification [30]. Blocking primers bind to predator DNA – and either stop universal primers from binding, or halt DNA polymerase from creating copies of predator DNA. A lobster-specific blocking primer was designed and tested for this study to allow better amplification of prey DNA, so that metabarcoding could better reveal aspects of the postlarval lobster diet.

The second eDNA technique discussed is real-time PCR (rtPCR). This approach is sometimes called ‘quantitative PCR’ (qPCR). Here we use the phrase ‘rtPCR’ as we highlight the real-time nature of this approach rather than its potential quantitative aspects. One advantage of rtPCR over metabarcoding is that no DNA sequencing is required, making it cost effective and providing a faster turnaround time. However, the rtPCR assay is taxon specific, making it appropriate for targeted identification of diet constituents rather than broad characterization of a predator’s diet. The key feature of rtPCR is use of DNA primers and a fluorescently-labeled DNA probe, specifically designed to target and detect a DNA sequence unique to the taxa of interest. As target DNA amplification continues, the level of fluorescence increases, and fluorescence above a threshold value constitutes a positive detection [31]. The number of PCR cycles necessary to amplify beyond the threshold is called the ‘Cycle quantification’ value (Cq), which can be used to compare the relative rate at which different samples amplify. Due to evidence of the importance of *C. finmarchicus* to the survival pelagic lobster stages [16], we designed our rtPCR assay to detect this species in the diet of lobster postlarvae.

This is the first time these molecular techniques have been applied to the postlarval lobster diet. Here, we not only describe in-depth methodological considerations so that others may apply these techniques; we also compare and contrast their use against one another and conventional microscopy. Mixed-methods approaches to diet analysis are rare; our analysis of postlarval lobster diet suggests it should be more widely adopted, to better represent the trophic status of the taxon in question.

## Methods

All specimen collections were conducted under special license number 2022-40-01 issued by the Maine Department of Marine Resources.

### Larval collection

Larvae for this study were collected from the University of Maine’s research vessel R/V Ira C. through a combination of horizontal tows at depth and neuston tows at the sea surface. Horizontal tows were conducted with 1 m diameter ring nets with a mesh size of 1000 µm and a cod end mesh size of 1000 µm. Neuston tows were conducted with a 1 m x 2 m rectangular neuston net with a mesh size of 500 µm and cod end size of 1000 µm. These tows were conducted at several stations near the mouth of the Damariscotta River, Maine (USA). Once brought aboard, larvae were sorted by developmental stage and placed into separate vials in a cooler, then transferred to a −80 °C freezer back at the lab. Additional lobster larvae were provided by the Maine Department of Marine Resources (DMR) collected in the same area, and by Normandeau Associates, Inc. from the coast of New Hampshire. Larvae supplied by Normandeau Associates and Maine DMR were stored in 95% ethanol and refrigerated. While larvae were collected at all four pelagic stages of development, only postlarvae were evaluated in this study due to their greater availability across all sampling events and their larger size. During this study, we dissected a total of 112 postlarvae for microscopy, 56 postlarvae for metabarcoding, and 48 postlarvae for rtPCR. Only postlarvae with visibly full guts (as evidenced by the presence of a dark bolus) were chosen for metabarcoding to increase the odds of successful prey amplification. This is the first application of molecular-based gut content analysis with postlarval American lobsters.

### Visual inspection by microscopy

Postlarvae stored in ethanol were rinsed in distilled water and then dissected to remove the foregut (cardiac and pyloric stomach). To do so, the head of the postlarva was removed from the body just behind the eyestalks with a pair of fine dissecting scissors to sever the esophagus and mouthparts from the cardiac stomach, as well as to remove some of the connective tissue of the carapace. The carapace was then gently removed from the body and the foregut excised and then transferred to filtered seawater to remove extraneous tissue. Seawater filtered through a 0.2 µm cartridge filter was used at this rinsing step to negate the osmotic impact of distilled water, which causes stomach tissue to swell, flake off, and mix with the stomach contents.

**Fig 1:**
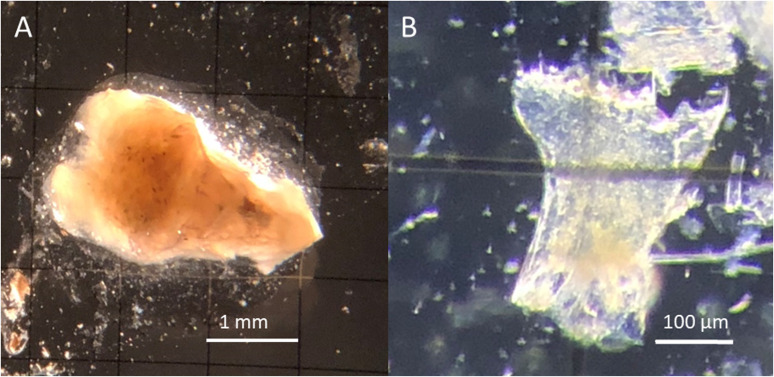
Example of a dissected postlarval lobster foregut and typical “hard part” used to identify prey. (A) Foregut of a postlarval lobster on a 1 x 1 mm gridded Sedgwick rafter. (B) *Calanus sp.* mandibular gnathobase isolated from postlarval gut.

The stomach was placed on a 1 x 1 mm gridded Sedgwick rafter slide and then pulled open with a pair of forceps (Fig 1). The stomach contents were rinsed out with dilute Lugol’s solution (<1%) to increase contrast of the prey, and the slide was then flooded with Lugol’s solution. The gut contents were distributed across the slide for identification by microscopy (Olympus SZX16). The gridded slide provided a valuable size reference in the identification of gut contents. Presence/absence of diet constituents was recorded to the lowest possible taxonomic unit. The percent frequency of food categories was then ranked among the sample of postlarvae examined. Methods used for prey identification included consultation with taxonomic keys and experts, survey of potential diet constituents from the same and concurrent net tows with finer mesh, and close anatomical examination of potential prey. Diet particles that could not be identified immediately were photographed for later identification.

### DNA extraction

A major motivation for considering DNA-based methods for diet analysis is the time required to build competency to identify diet constituents by visual methods. Postlarvae for analysis by eDNA methods were dissected with a similar technique to the one described above, with a few alterations to minimize cross-contamination between samples. All tools used for dissection were dipped in ethanol and sterilized over a Bunsen burner in between samples. Postlarvae were dissected over a fresh, sterile petri dish. Once the gut was removed, it was placed directly into a 1.5 mL DNA LoBind microcentrifuge tube (Eppendorf). If the gut was not extracted for DNA immediately, it was submerged in DNA/RNA Shield (Zymo Research), and placed in a −20 °C freezer until extraction.

The ChargeSwitch Forensic DNA extraction kit (Invitrogen) was used for DNA extraction as this kit has been demonstrated to be adept at recovering degraded or rare DNA, which fits the profile of prey DNA recovered from a predator’s stomach [10]. Extractions were conducted per the manufacturer’s directions with one alteration: digestion of the sample with Proteinase K was conducted for 2 hours rather than 1 h. Additionally, after the first hour of lysis the sample was ground with a sterile pestle to ensure complete lysis of all gut contents. A high-sensitivity Qubit DNA concentration assay (Invitrogen) was conducted to determine whether extractions resulted in measurable DNA.

### Development and testing of lobster blocking primer

To reduce the contribution of predator DNA to metabarcode sequencing results, samples used in diet studies often need to be amplified in the presence of a predator-specific blocking primer. There are a variety of different blocking primer types (summarized by Vestheim and Jarman [30]), and typically blockers which arrest PCR prior to primer annealing are most successful. However, we developed a blocking primer that halts the elongation of lobster DNA, as there was no lobster-specific sequence region that was close enough to the universal 18S priming sites to utilize the ‘arresting’ approach [30].

Lobster postlarvae provide a particularly challenging case study as their diet is composed chiefly of other crustaceans [32–34], leading to a high degree of genetic similarity between predator and prey, even within the variable barcode region of the 18S rRNA gene. In preliminary tests, standard blocking primers were ineffective, and had the tendency either to allow amplification of lobster DNA, or hinder the amplification of both host and prey DNA. To surmount this issue a Peptide Nucleic Acid (PNA) version of the blocking primer was utilized. A PNA is a synthesized molecule similar in structure to DNA, but lacking a charged phosphate group [35]. Because of this, a PNA-DNA dimer has a stronger bond than a DNA-DNA dimer, as it lacks an opposing electrostatic force. This quality makes it possible to design very short blocking primers that 1) bond strongly and accurately to the targeted DNA region, and 2) have a greater degree of specificity as there will be fewer opportunities for sequences to match up with non-target DNA. PNA molecules also have higher melt temperatures (Tm), the temperature at which an oligonucleotide will most efficiently bind to single stranded DNA, than standard oligonucleotides. The higher Tm allows for a PCR with 2-step annealing following the high temperature denaturing step. The higher temperature first annealing step allows the blocking primer to attach to the single stranded predator DNA before the metabarcoding primers begin to anneal, leading to better specificity of the blocking primer and greater suppression of the predator DNA. The PNA was synthesized by PNA Bio, Inc. (Thousand Oaks, CA) and was resuspended to 100 µM with molecular-grade water (warmed to 55 °C) upon receipt.

Lobster DNA was isolated from an ethanol-preserved postlarval specimen for use in testing the PNA blocker. Lobster DNA was extracted using the methods described above, and then tested for amplification using 18S V4 universal metabarcode primers [36]. The lobster PCR product was then isolated by gel electrophoresis for cloning into the pCR4-TOPO cloning vector (ThermoFisher Scientific). The resulting plasmid DNA product was diluted into a 10 ng/µL stock solution that was used for all subsequent testing.

#### PNA Blocker concentration.

The effect of the PNA blocker concentration on its efficacy of blocking lobster amplification during PCR was tested over PNA concentrations that spanned two orders of magnitude. The blocker was tested at concentrations of 0.25, 0.5, 2, 5, 10, 15, and 20 µM per reaction across three different trials. Each concentration was tested in triplicate, and a blank No Template Control (NTC) was included for each concentration. A sample containing lobster DNA but no PNA (0 µM) was also included in triplicate for each trial as a point of comparison. The samples without blocker served as a benchmark for determination of how many PCR cycles each blocker concentration delayed amplification. Reactions were run as an rtPCR using a Bio-Rad CFX96 thermocycler in order to visualize results in real-time. Each reaction contained varying volumes of the PNA, DNA-free water, and either 2 µL of lobster DNA (10 ng/µL) or DNA-free water for the NTC. Aliquots of the PNA were heated to 80 °C prior to use in PCR, per the manufacturer’s instructions. PCR reaction conditions included: Primers E572F- CYGCGGTAATTCCAGCTC (500 nM) and E1009R- AYGGTATCTRATCRTCTTYG (500 nM), SYTO-9 DNA binding dye (500 nM), 1x Phusion Master Mix (New England Biolabs), and DNA-free water to 25 µL. Thermal cycling included an initial incubation at 98 °C for 30 sec., followed by 40 cycles of 98 °C for 10 seconds (denature), 75 °C for 30 seconds (PNA annealing), 55 °C for 30 sec. (primer annealing), and 72 °C for 30 sec. (DNA polymerase extension).

#### Cross-reactivity.

The use of a PCR blocker requires testing to ensure its specificity and that it does not also block amplification of prey DNA, especially where predator and prey are closely related. *In silico* testing with Geneious v9 (Biomatters Ltd.) suggested that the lobster-blocking PNA was specific to *Homarus americanus*. However, the impact of the blocker on amplification of one common zooplankton species (the copepod *C. finmarchicus*) and one common algal species (the diatom *Pseudo-nitzschia* sp.) was tested at the bench to ensure PNA efficacy. Genomic DNA was isolated from each of these organisms in the method described above, diluted to concentrations of 10 ng/µL and mixed with lobster DNA in order to determine whether the blocker would be successful in blocking the lobster DNA from amplification while allowing the ‘prey’ DNA to amplify. These mixtures were tested in triplicate to see how well they amplified, with and without the blocking primer in comparison to reactions containing lobster DNA alone. Additionally, DNA was extracted from the gut of a lab-reared postlarva known to have grazed on *C. finmarchicus* exclusively, to provide a more natural test of the blocker. A field collected postlarval gut sample was also included for comparison.

#### Annealing temperature.

The annealing temperature of the blocker was estimated to be 70 °C when synthesized by PNA Bio Inc. (Thousand Oaks, CA). It is recommended to use an annealing temperature 5 °C above this value for greater specificity. In order to determine whether 75 °C was indeed the optimal temperature, a gradient PCR test was performed with annealing temperatures of 70, 75, and 78 °C. At each temperature, controls without any blocker were run in triplicate, as well as triplicate 25 µL reactions containing 2 µM blocker plus a No Template Control (NTC) reaction.

### DNA Metabarcoding

Universal DNA metabarcoding primers targeting the V4 region of the 18S rRNA gene were used, as described in Comeau et al. (2011), and were selected for the broad coverage of eukaryotic taxa exhibited by these primers. The optimal PNA concentration in each reaction was 2 µM, based on the previously described testing to determine the minimum concentration of PNA needed for maximum blocking. Amplification of lobster prey was performed with the 18S primers, the novel lobster-blocking PNA and high-fidelity Phusion Hot Start 2X mastermix (New England Biolabs) with the same thermal protocol as listed above. The resulting PCR products were concentrated with the Zymo-5 Clean and Concentrator kit (Zymo Research) and the concentration of DNA present in each sample was determined using a Qubit fluorometer and the high sensitivity dsDNA quantification reagents (Invitrogen). Of the 56 postlarvae dissected for metabarcoding, 45 were grouped into 9 samples containing 5 guts each to test whether grouping guts increases the concentration and detection of prey DNA. Additionally, of the remaining 11 single gut samples, the first three were pre-amplified in the lab with the PNA. The remainder were sent for sequencing along with an aliquot of the PNA to be included during library prep at the sequencing facility; this minimized the total rounds of PCR for each sample, reducing PCR errors and the potential for amplification of predator DNA. PCR for 5 of the grouped (= 25 postlarvae) and 3 of the individual gut samples (totaling 28 postlarvae) failed to amplify prey, leaving 28 postlarval guts among 12 samples – including 4 grouped (= 20 postlarvae) plus 8 individuals. A flowchart depicting this sampling scheme can be found within the supplementary data (S1 Fig). Sequencing was conducted at Dalhousie University’s Integrated Microbiome Resource (IMR) laboratory in Halifax, Nova Scotia, Canada. As amplification through PCR can introduce errors that may be misinterpreted as true biological variation, reads must be assessed for quality using bioinformatics tools. This is typically accomplished through algorithms designed to filter the raw reads, managing error rates and inferring true biological diversity through clustering reads into Operational Taxonomic Units (OTUs), or Amplicon Sequence Variants (ASVs) [37,38]. An OTU is a cluster of reads based either by similarity (typically 3%), or through comparison to a reference library. An ASV method infers biological reality rather than artificial clusters through the expectation that “true” sequences are more likely to be repeated within a sample, and can distinguish unique ASVs based on a single nucleotide difference [37]. This technique, utilizing the DADA2 (Divisive Amplicon Denoising Algorithm) bioinformatics pipeline is capable of resolving fine-scale biological variation, and performs as well or better than similar OTU methods [37,38]. Amplicon Sequence Variants (ASVs) were inferred from the sequence reads using the DADA2 bioinformatics pipeline [38], and taxonomy assignments were produced using the SILVA reference database [39]. Any ASVs attributed to lobster were removed to focus solely on inferred prey ASVs. After filtering data to just those ASVs which represented likely prey items, Relative Read Abundance (RRA)- the proportion of reads attributed to each individual ASV in a sample, was calculated using the following formula: $\frac{\#of\;reads\;for\;an\;ASV}{Total\;\#\;of\;reads\;in\;a\;sample}$. It should be noted that cannibalism would be undetectable using the eDNA metabarcoding approach. The iNext package in R [40] was used to extrapolate and plot the relationship between number of postlarval guts sampled and metazoan prey diversity comparing metabarcoding and microscopy data. A comparison of the broad prey categories identified by each method was also conducted. To provide a more statistically robust comparison of the microscopy dataset (n = 112 postlarvae) to the much smaller metabarcoding dataset (n = 28; 8 single guts + 20 guts grouped into four samples of 5 guts each), we used a bootstrapping approach to randomly sample 28 postlarvae 1000 times from the pool of 112 postlarvae analyzed by microscopy dataset to generate a mean and 95% confidence interval.

### Real-time PCR (rtPCR)

*Calanus finmarchicus* specific primers and a Minor Grove Binding (MGB) probe were designed to target a species-specific region of the cytochrome oxidase subunit-I marker gene (COI) from this specific lobster-diet prey species. PCR reaction conditions included: Primers Cal_fin_COI_Fwd- AATGCTATTGGACCGTATG (500 nm), Cal_fin_COI_Rev- TCTGTTAATAGTATCGTGATAGC (500 nm), the probe Cal_fin_COI_MGB- TCATCACTGCTGTC (250 nm), 1X IDT PrimeTime Master Mix (Integrated DNA Technologies), and DNA-free water to 25 µL. Thermal cycling included an initial incubation at 95°C for 3 min., followed by 45 cycles of 95 °C for 15 seconds (denature), and 60 °C for 30 seconds (primer and probe annealing and extension). The MGB probe was 5’-labeled with FAM and 3’-labeled with MGB Eclipse quencher (Eurofins Genomics). A lab-reared postlarval lobster, fed a known diet of *Calanus*, was tested with this rtPCR assay to demonstrate that the assay was capable of detecting *Calanus finmarchicus* within the gut of a postlarval lobster. A total of 48 field-caught postlarvae were assessed using the rtPCR assay. All rtPCR reactions were run in triplicate on a Bio-Rad CFX96 thermocycler. Any samples that amplified above a fluorescence threshold determined by the CFX Manager software were considered to have tested positive for the presence of *C. finmarchicus*.

## Results

The results described here focus on the utility and metadata for each technique presented. For a more detailed discussion of the prey taxa identified through these methods, see Ascher et al. (2023) and forthcoming works.

### Microscopy

In total, 7 metazoan phyla, including 9 classes, 5 orders, 5 families, and 5 genera were detected as postlarval lobster diet constituents after dissecting and investigating 112 lobster postlarvae under a microscope. The greatest proportion of prey identified by microscopy were recorded at the class level (40%), with a steep decline in identifications between class and order (13%). The vast majority of identified prey were arthropods due to their hard parts (such as carapace, mandibles, etc.) that were better preserved (Fig 1). Common prey included copepods, malacostracans, and polychaetes, which were present in 50.0%, 55.4%, and 9.8% of postlarvae, respectively. There were no unique identifications made at the family level. The only families of diet constituents recorded were inferred from the presence of a prey taxa within a representative genus.

### Development and testing of lobster blocking primer

#### PNA blocker concentration.

Without any blocking primer, the lobster DNA sample amplified after an average of 15.44 cycles (Table 1). The number of cycles until amplification can be used to calculate the effectiveness of the blocker, denoted as delta Cq or△Cq – the difference in amplification cycle number with the PNA blocker present compared to the cycle number without the PNA blocker. At PNA blocker concentrations of 0.25 µM and 0.5 µM, the blocking efficacy was roughly similar with a delay of about 7 Cq, while increasing concentration to 2 µM provided a jump in blocking efficiency, with a delay of more than 12 Cq. The highest ΔCq (greatest delay) was recorded at a PNA concentration of 10 µM, however at that concentration the ΔCq exhibited greater variation (minimum ΔCq of 4.21, and a maximum of 22.76) compared to other blocker concentrations (Table 1). Increasing PNA concentration beyond 10 µM decreased blocking efficiency slightly.

**Table 1:** Technical replicates (n), mean Cq, mean ΔCq, and standard deviation for each PNA blocker concentration.

PNA concentration (µM)	n	Mean Cq	Mean ΔCq	Std Dev
0	9	15.44	NA	2.18
0.25	3	26.03	7.73	0.26
0.5	3	25.69	7.39	0.29
2	6	25.48	9.21	0.88
5	3	22.36	8.56	0.07
10	6	26.50	12.48	6.49
15	6	22.68	8.67	1.16
20	3	21.90	7.66	0.39

Mean ΔCq is the average difference between each PNA concentration and the control within each respective PCR, rather than the difference between final mean Cq values to control for any variation between trials.

#### PNA cross-reactivity.

**Fig 2:**
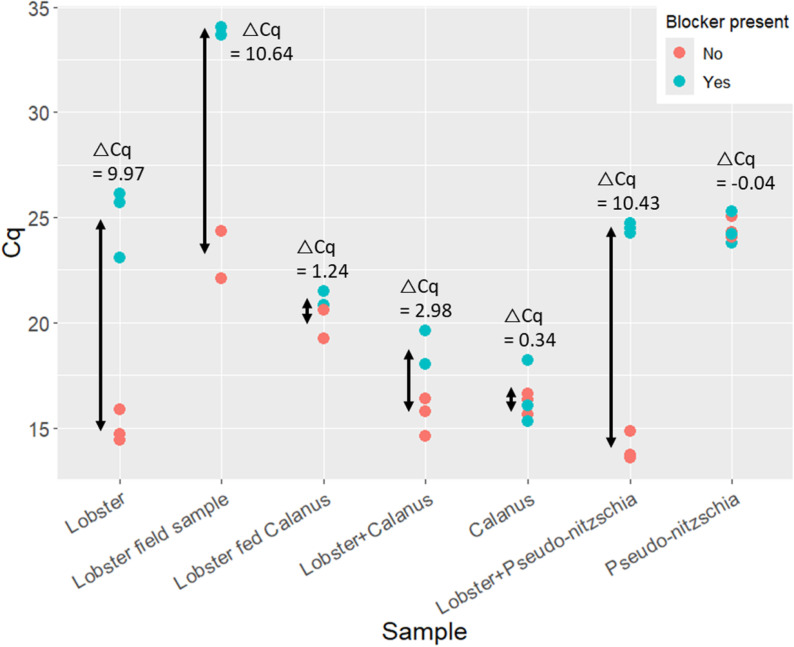
Cycle number (Cq) at which different samples amplified with and without the PNA blocker present. Samples included single-source genomic DNA templates as well as mixtures of predator (lobster) and prey (*Calanus* or *Pseudo-nitzschia*) genomic DNA to test the cross-reactivity of the PNA blocker. A lab-reared larva fed *Calanus* as well as a field-caught lobster larval sample were also tested. Arrows provide a visual representation of ΔCq.

Lobster DNA alone amplified after an average of 15.00 cycles and was delayed by 9.97 cycles when the blocker was added at a concentration of 2 µM. With no blocker present samples containing a mixture of lobster and prey DNA amplified after a similar number of cycles; while the lab-reared postlarva fed on *Calanus* and the field-caught postlarva each amplified later than the lobster DNA alone. The *Pseudo-nitzschia* algal sample amplified the latest, while the Cq of *Calanus* was roughly similar to that of lobster at 16.19 (Fig 2). Addition of the blocker had no significant effect on the amplification of *Calanus* or algal samples alone – demonstrating that the lobster-specific blocker does not inhibit amplification of these potential prey species. However, the reactions with the lobster blocker did delay amplification in samples containing a mixture of lobster and prey, presumably impacting the amount of lobster DNA amplified. DNA amplification of the field-caught lobster postlarvae, with an unknown feeding history, was delayed by similar amounts as the postlarvae with added algal DNA. Importantly, we observed an intermediate delay when lobster DNA was mixed with *Calanus* DNA, both when lab-reared postlarvae fed on *Calanus*, and when lobster DNA was deliberately mixed with *Calanus* DNA. We interpret these outcomes in the discussion.

#### Annealing temperature effects.

**Fig 3:**
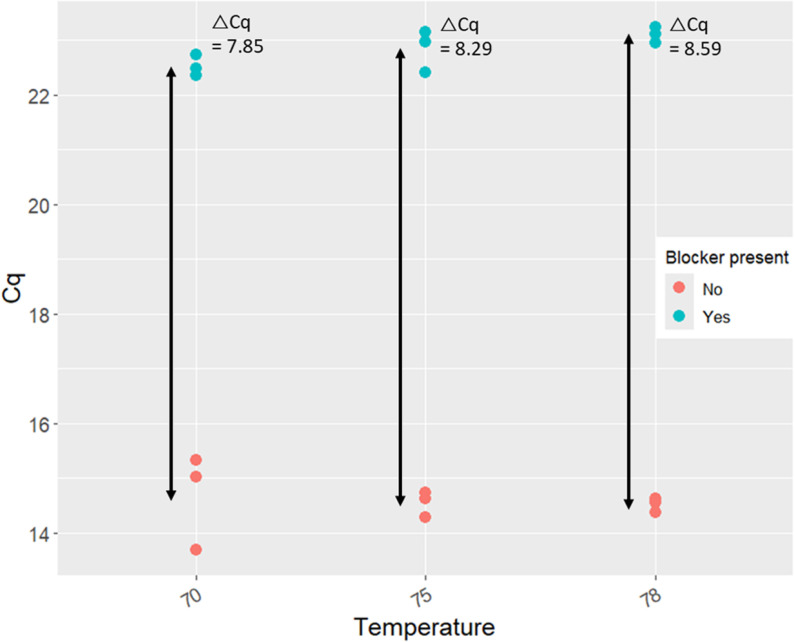
Amplification of lobster DNA at 3 different annealing temperatures with and without PNA blocker present. Cq values were recorded for amplification using 18S V4 metabarcoding primers. An ANOVA found no significant effect of temperature on Cq value with (F_2,9_ = 3.68; p = 0.091, R^2^ = 0.55) or without (F_2,9_ = 0.081; p = 0.924, R^2^ = 0.03) the blocker present. Arrows provide a visual representation of ΔCq.

In general, there was little change in blocking primer efficiency over the range of annealing temperatures tested (70–78 C). Without the blocker, we found no significant effect of temperature on Cq (ANOVA *F*_*2,9*_* = 0.081; p = 0.924, R*^*2*^* = 0.03*). Similarly, with the blocker present at 2 µM, Cq did not vary significantly (*F*_*2,9*_* = 3.68; p = 0.091, R*^*2*^* = 0.55*) (Fig 3). Correspondingly, ΔCq appeared to increase slightly with increased temperature, but not to a significant degree (*F*_*2,9*_* = 1.43; p = 0.310, R*^*2*^* = 0.32*).

### Metabarcoding

**Fig 4:**
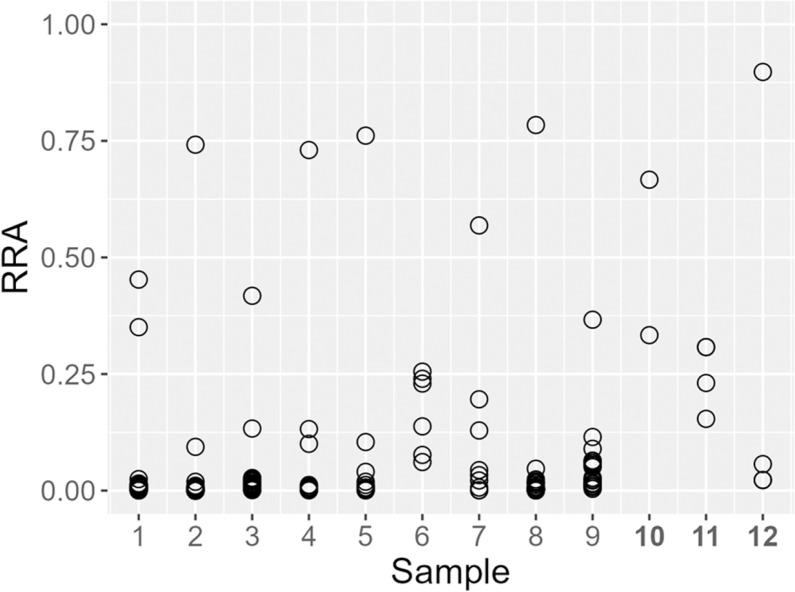
Relative Read Abundance (RRA) for each ASV within a sample. Each dot represents a single ASV, and RRA was calculated after removal of ASVs attributed to lobster. Samples in bold represent the 4 grouped samples, containing five postlarval guts each. Samples in bold represent the 4 grouped samples, containing five postlarval guts each.

**Fig 5:**
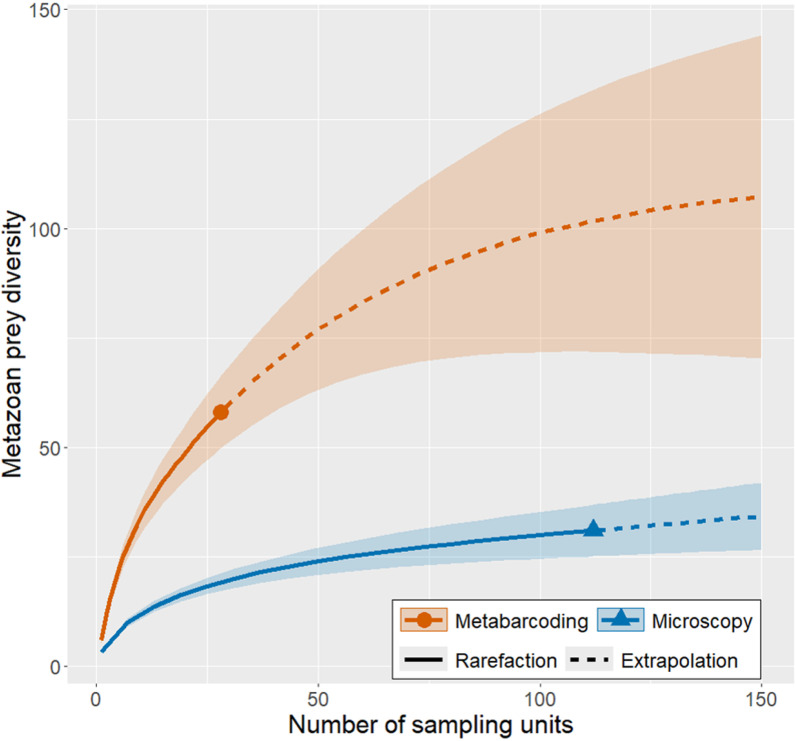
Relationship between postlarvae sampled (sampling units) and metazoan prey diversity using metabarcoding and microscopy.

**Fig 6:**
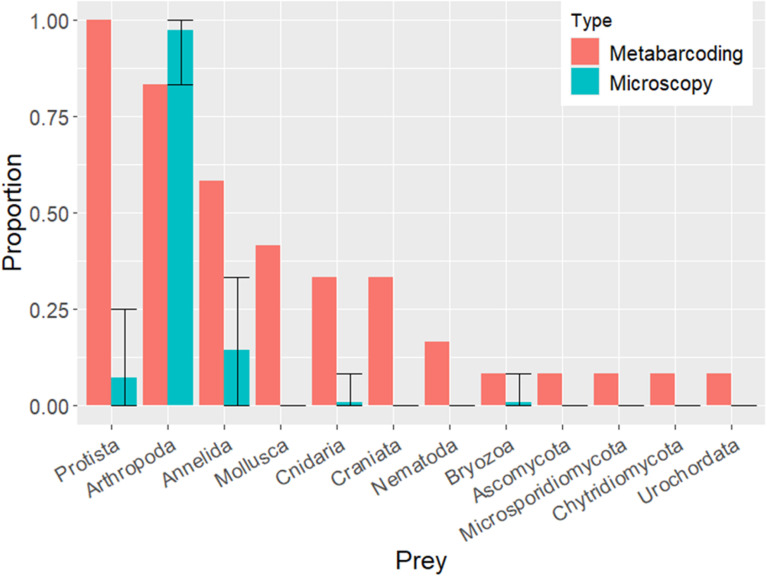
Proportion of field-collected postlarvae containing broad prey categories identified through metabarcoding and microscopy. Metabarcoding was conducted on a sample of 12 postlarvae. For postlarvae analyzed by microscopy we generated a mean proportion (+95% CI) by bootstrap sampling of 12 random postlarvae drawn 1000 times with replacement from the total pool of 112 postlarvae analyzed.

Of the postlarval gut samples submitted for sequencing, 12 were successfully amplified for prey DNA; eight of these were single gut samples while four were grouped samples containing five guts each. Grouping guts together was meant to increase the concentration of prey DNA and therefore enhance the odds of successfully amplifying and sequencing prey. However, grouped samples did not appear to provide any benefits to prey detection. From the samples which amplified successfully, a total of 1668 distinct ASVs were inferred from 2,206,103 reads. Of these, 745 ASVs were attributed to either *Homarus americanus,* or the Norwegian lobster *Nephrops norvegicus*. As the Norwegian lobster is not present in the Gulf of Maine, these ASVs are almost certainly misidentified American lobster DNA due to *Nephrops* and *Homarus* being nearly identical at this genetic locus. These ASVs were removed from further consideration as they most likely represent lobster DNA that was not fully blocked. In Fig 5 and 6 we primarily considered metazoan ASVs evident in the diet, although Fig 6 includes protists for comparison. We also removed any ASVs relating to unicellular organisms (likely derived from secondary consumption), or other organisms which clearly were not diet constituents (e.g., terrestrial plants), leaving 328 prey ASVs inferred from 241,454 reads. Taxonomic assignments using the SILVA 18S rRNA database [39] as reference revealed some similar taxa as identified through microscopy, but also included some ‘soft-bodied’ prey-items that would be more difficult to identify by microscopy. The total number of both metazoan ASVs and sequence reads were unevenly distributed among samples with some samples containing dozens of ASVs and thousands of reads, and others containing just a few of each (Table 2). A little under half (146) of these ASVs could be identified down to a putative species given taxonomic limitations of the 18S V4 region. Most ASVs had relatively low RRAs, with each sample typically containing 1–2 ASVs which appeared significantly more abundant relative to others within the sample (Fig 4).

**Table 2:** Total reads and ASVs after removal of lobster ASVs in the 12 postlarval metabarcoding samples.

Sample #	1	2	3	4	5	6	7	8	9	10	11	12	Total
ASVs	32	145	49	12	20	6	9	28	17	2	4	4	328
Reads	6603	198329	5266	3078	8412	196	4831	13930	513	6	26	264	241454

Metabarcoding also revealed a greater diversity of prey taxa per sample when compared to microscopy (Fig 5). While microscopy revealed only about 25 unique metazoan taxa (ranging from species to phylum) in the diets of 112 postlarvae, metabarcoding identified more than 50 unique metazoan categories from only 12 samples (representing 28 postlarvae due to the grouping of some individuals). Broadly, metazoan prey identified through metabarcoding were distributed among a greater number of phyla compared to microscopy. Although the two methods consistently found the majority of postlarvae consumed arthropod prey, it is noteworthy that metabarcoding detected soft-bodied or single-celled categories considerably more often than did microscopy (Fig 6). Based on the number of samples processed, projection of the rarefaction curves in Fig 5 suggests there is potential for much greater taxonomic resolution with metabarcoding than with microscopy.

### rtPCR

**Fig 7:**
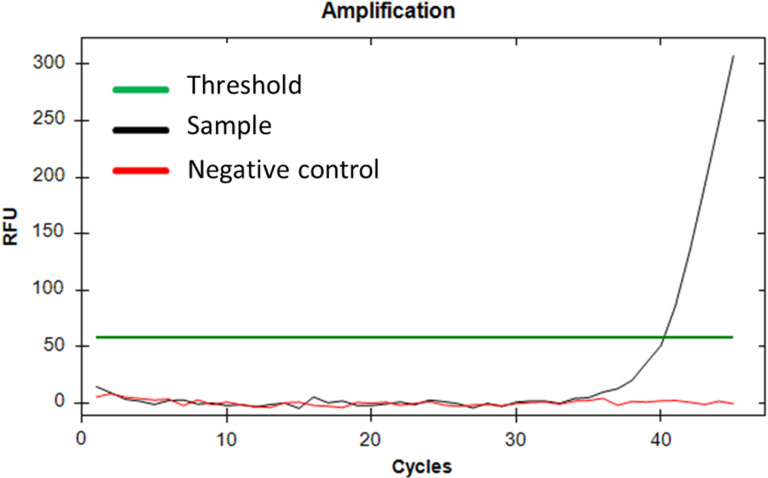
Detection of *Calanus finmarchicus* using rtPCR of DNA extracted from a postlarva fed *Calanus finmarchicus* versus a negative control containing no *C. finmarchicus.* The late amplification is consistent with the very small amounts of prey DNA expected from a single postlarva feeding exclusively on *Calanus finmarchicus*.

Testing a lab-reared postlarva known to have consumed *Calanus* demonstrated that the rtPCR assay was capable of amplifying *Calanus* DNA within a postlarval lobster gut (Fig 7). Subsequent testing of field-caught lobster postlarvae with unknown feeding history revealed that 10 out of 48 samples amplified with our *Calanus finmarchicus* rtPCR assay, although this amplification occurred relatively late. Of the 10 samples with *Calanus* present, an average of 1.9 of the three technical replicates amplified (Table 3). Despite the late amplification, the rtPCR approach revealed that 20.8% of the postlarvae had consumed *Calanus*, and demonstrated that the rtPCR primers and probe designed for this study were effective on field-caught postlarval samples.

**Table 3:** Metadata for the 10 postlarvae containing *Calanus finmarchicus.*

Sample #	Average Cq	Lowest Cq	Highest Cq	# of amplified replicates
1	42.00	42.00	42.00	1
2	36.88	27.92	41.67	3
3	34.06	25.54	38.75	3
4	39.46	35.69	42.66	3
5	37.21	35.52	38.90	2
6	39.19	36.03	42.34	2
7	37.52	37.52	37.52	1
8	39.49	39.49	39.49	1
9	39.21	37.34	41.07	2
10	40.40	40.40	40.40	1
Average	38.54	35.75	40.48	1.9

The Cq value represents the cycle number at which a sample amplified beyond a baseline threshold, indicating presence of *Calanus finmarchicus*.

## Discussion

The molecular methods discussed here represent the first application of DNA-based approaches applied to the postlarval lobster diet. These data are likely a snapshot of the totality of the postlarval lobster diet, but provide guidance for future DNA-based studies that should become easier, faster, and more cost effective with continuing technological improvements. The DNA sequencing approach employed was successful for evaluating the diets of lobster postlarvae, and revealed additional prey taxa beyond those confirmed by microscopy. This clear advantage of DNA sequencing is that it identified prey types often overlooked in microscopy, and provided high, often species-level, prey resolution. However, the limitations of this approach are its semi-quantitative nature, possible taxon-skew in PCR amplification, and the potential for contamination. Thus, information derived from DNA sequencing is most valuable when combined with existing understanding of lobster postlarval ecology. Microscopy identified almost exclusively metazoan taxa, and only those with readily visible hard parts (e.g., crustaceans, mollusks, polychaeta, etc.), whereas metabarcoding identified a broader suite of metazoans, including gelatinous forms (cnidarians, etc.), as well as a host of protists (microalgae, foraminifera, etc.) that may have been consumed directly or were associated with the gut contents of metazoan prey, but would not be readily detectable by microscopy (Fig 6). It is interesting to note that these protistan sequences may provide insight into the identity of primary producers and/or primary consumers that link postlarval lobsters to the base of the marine food-web. Thus, the high frequency of protists and diversity of other taxa detected by metabarcoding, but not by microscopy, provide a completely new perspective on the larval diet. Analysis of diet constituents in greater detail will be included in a subsequent paper that is currently in preparation.

Though microscopy identified a narrower range of prey, it also required the fewest specialized materials or techniques and proved to be capable of taxonomically resolving prey to basic phylogenetic categories. However, the lack of more specific identifications illustrated the need for a high degree of taxonomic expertise using this technique. Metabarcoding was successful in providing some of those more specific identifications, but this technique was also the most expensive, and required development and testing of a novel blocking primer to mask ubiquitous predator DNA. Finally, rtPCR successfully targeted a prey species of interest, and demonstrated the potential for species-specific assays to reveal key portions of the diet that might otherwise go unnoticed. However, the species-specific rtPCR approach is not practical with currently available analytical platforms for revealing the full range of the postlarval diet.

### Microscopy

In general, microscopy is the most accessible method and has the longest history in diet analysis. Previous attempts to document the lobster postlarval diet were conducted using microscopy, including Juinio and Cobb [33] as well as Harding et al. [34]. These studies also recorded copepods and malacostracans as the most common prey of postlarvae, highlighting that similar methods tend to obtain similar results. Some differences are worth highlighting, however. For instance, each of these previous studies detected fish eggs in the postlarval lobster diet [33,34], a prey category absent from our microscopy dataset, although we detected fish DNA by metabarcoding. This serves to highlight how microscopy studies may vary due to individual biases, while molecular tools are more objective. However, without the context provided by the previous microscopy studies, it would be unclear whether the fish DNA detected through metabarcoding came from larvae, or environmental contamination, rather than eggs. Microscopy thus affords the opportunity to record more information on the disposition of gut contents than either of the eDNA techniques discussed. For example, 1) Many marine invertebrates have a variety of larval and adult forms, some of which may be more energy dense, and so provide a higher quality prey item for the predator. Depending on their taxonomic expertise, a researcher may be able to determine the larval form present in the gut, while eDNA would only be able to provide a taxonomic identification; 2) Some organisms found in the gut are not prey- a freshly dissected gut in this study revealed a nematode which was presumed to be a parasite rather than prey as it was observed to still be living; 3) Level of digestion of a prey item could be crucial information revealing how recently it was consumed [33]; and 4) Volumetric, gravimetric, or numerical abundance can help to reveal important information about the relative abundance of prey in the diet [22]. Finally, 5) Evidence of cannibalism could be uncovered through microscopy, but would likely be missed using eDNA techniques [2].

In this study, presence of prey items was recorded, but not quantified further. Crustaceans tend to tear up their food rather than consuming prey whole, making it difficult to record the abundance of prey items, unless the presence of a single body part enables the enumeration of prey [6], which poses a challenge in its own right [5]. Approximately 40% of the microscopy data in this study could only be identified to the class level, and no prey were identified to the species level. In this study prey could only be identified as low as the genus level, and of the 20 prey items for which genus level data were recorded, 17 were copepods. This is worth noting as copepods have a notable diagnostic feature: the mandibles of copepods have distinct morphology allowing identification of a specific genus when present (Fig 1). This draws attention to a possible bias of microscopy; it is difficult to determine whether copepod genera were identified more often than other genera because of their abundance as prey, or the ease with which they can be identified. What’s more, using microscopy constrains the researcher to recording only that portion of the diet with identifiable hard parts. If the predator were to consume soft-bodied organisms, such as ctenophores, urochordates, or cnidarians, they would be unlikely to be identified at all by microscopy [5].

### Metabarcoding

Metabarcoding with universal eukaryote PCR primers is one way to complement microscopy. Where microscopy may be open to significant bias based upon researcher taxonomic expertise, metabarcoding is a technique that, if executed properly, any researcher should be able to obtain similar data from the same sample. Additionally, samples are not destroyed after analysis as happens during microscopy, allowing for processing of multiple replicates from the same sample to increase confidence. Use of the lobster-specific PNA blocking primer was critical for application of the metabarcoding method. One challenge with development of the lobster-blocking PNA for this study was trying to find a unique lobster-specific sequence within the relatively short V4 region of the 18S rRNA gene targeted by our DNA metabarcoding approach. Focusing metabarcoding efforts on this region was a trade-off between potentially discovering a wide taxonomic diversity of postlarval lobster prey based on the size and breadth of 18S reference databases such as SILVA [39] and PR2 [41], but possibly missing the detection of prey that were more genetically similar to lobsters (e.g., other crustaceans) due to the relatively conserved nature of the 18S rRNA gene. Newer ‘long-read’ metabarcoding approaches may alleviate this current challenge, by providing more options for developing alternative PNAs. Targeting additional loci commonly used in metabarcoding, such as COI, may also be beneficial as different markers and their reference databases can be more complete for different taxa.

Despite these challenges, our blocking primer was capable of significantly suppressing the amplification of lobster DNA while having only a mild effect on the amplification of potential prey species (Fig 2). However, no change to concentration or annealing temperature could suppress the amplification of lobster DNA completely (Table 1, Fig 3). While increased concentration of a blocking primer is typically associated with greater blocking efficacy [12,30], increased concentration did not have a particularly dramatic effect on the efficacy of the PNA blocking primer tested here (Table 1). This is likely because at higher concentrations (>5 µM) PNA oligonucleotides tend to have difficulty remaining in solution, and so the higher PNA concentrations had less of an effect on the suppression of PCR for lobster DNA (PNA Bio, personal communication, 2022). As a result, 2 µM was chosen as the optimal blocker concentration, despite the greater (but more variable) average blocking effect of the 10 µM PNA concentration. Blocking primers need to be as uniformly reliable as possible; adding the blocking primer at a concentration of 10 µM led to significant variability in efficiency, likely due to differential solubility among samples (Table 1), and utilized five times the amount of PNA per reaction, adding significant cost per sample. Additionally, though the cross-reactivity of the blocking primer was examined, it is impossible to rule out the possibility that it would inhibit amplification of some prey species without more extensive testing. Therefore, it is best to use the lowest effective concentration of blocking primer [30]. As a result, 2 µM as the next most efficient blocking concentration was preferable to 10 µM.

Using the PNA blocker at a concentration of 2 µM, the blocker could suppress the amplification of lobster DNA in the presence of prey DNA without unduly affecting the amplification of the prey. Alone, amplification of DNA from the copepod *Calanus finmarchicus* and the alga *Pseudo-nitzschia sp.* was unaffected by addition of the blocker. When mixed with lobster DNA there appeared to be a slight effect on the amplification of *Calanus finmarchicus*, however that is more than likely due to the lobster and *Calanus* DNA competing for the same pool of primers; rather than an actual impact of the PNA blocker. This result explains the slight delay in amplification seen in the lobster + *Calanus* sample (Fig 2). The lobster + *Pseudo-nitzschia sp.* sample seemed to be significantly delayed by the addition of the PNA blocker, however this was also misleading. From the isolated *Pseudo-nitzschia sp.* sample it was evident that the 18S V4 primers tended to amplify this algal sample later than the other DNA targets. Therefore, in the mixed lobster+*Pseudo-nitzschia sp.* sample, the earlier amplification in the unblocked trial was likely due to the lobster DNA amplifying, and the later amplification in the blocked trial was likely a mixture of both lobster and *Pseudo-nitzschia sp.* amplification. Since no effect of the blocker on the pure *Pseudo-nitzschia sp.* sample was detected, it can be deduced that the PNA blocker was not significantly impacting its amplification. The small change in Cq between the blocked and unblocked trial for the lab-reared postlarva fed on *Calanus* was a promising result, as it demonstrated that the consumed *Calanus* was most likely amplifying prior to significant postlarval lobster amplification. However, the △Cq for the field-caught postlarval sample was similar to that of the pure lobster DNA, which could mean that diet DNA from field specimens may be more dilute or degraded when compared to the lab-reared postlarva known to have consumed *Calanus* within hours of being sampled (Fig 2). Variable concentration or quality of dietary DNA from field-caught postlarvae could lead to inconsistent blocker efficiency, as low concentrations of poor-quality DNA may require longer to amplify or more technical replicates to detect a positive test result.

Under ideal circumstances a blocking primer would fully suppress predator DNA amplification. While we did not get full blockage, our PNA blocker did delay amplification of lobster DNA by an average of 9.21 cycles. This translates into an approximately 600x lower (predicted) concentration of predator DNA in the final pool of PCR products. In addition, the degree of blocking was unaffected by either the annealing temperature or blocking primer concentration suggesting that the blocking primer is robust. Although a greater amount of suppression would be ideal, a moderate amount of suppression could be expected from this blocking primer in a variety of PCR conditions and a range of concentrations. A blocking primer designed to overlap with the priming region, rather than halt elongation, could have provided greater suppression. However, the high degree of relatedness between postlarvae and their prey precluded such a design as lobster DNA is not sufficiently unique nearby the priming locus. Designing the blocking primer to overlap the priming region could have led to suppression of prey species as well in this situation. On the other hand, the incomplete suppression demonstrated here may cause species-masking. Taxa to taxa, decisions regarding marker choice and blocking location must be balanced to provide the best possible suppression without impacting prey amplification. Full suppression may not always be achievable. Such instances serve to highlight the use of a mixed-methods approach, where microscopy and rtPCR can help fill in gaps. The PNA blocking primer protocol developed herein provides a good example for future studies to use as a jumping off point for their own sequencing-based diet studies. One recommendation to reduce predator co-amplification is to perform a single round of PCR amplification that includes blocking primer during the library preparation step. This procedure will reduce the total number of PCR cycles to which samples are subjected, thereby reducing the fraction of predator DNA in the pool of sequences. Samples 4–15 in this study were processed in this manner, as opposed to samples 1–3, which were pre-amplified with the PNA blocker in a first-round PCR (in-house), prior to a second-round PCR for library prep (at IMR) before sequencing. In comparison, samples 1–3 have a greater number of reads and ASVs (Table 2), however several rounds of sequencing using the pre-amplification method failed entirely before choosing to add the blocker during library prep. Additionally, these first three samples contained a significant number of unknown malacostracan ASVs which may represent unblocked lobster DNA. In comparison, samples 4–12 which were processed without the lab pre-amplification contained almost no unknown malacostracan ASVs, reflecting the reduction in predator amplification we predicted. Regardless of technique there was significant variation in the number of ASVs and reads associated with each sample (Table 2), likely reflecting differences in gut fullness and how recently postlarvae consumed prey prior to sampling. It is possible the pre-amplification method could offer a greater volume of data, but with so few samples processed by this method it is difficult to say, and the greater reliability gained from foregoing the pre-amplification is preferable.

Counterintuitively, grouping guts together did not appear to enhance prey identification (Table 2). This might be due to species masking from incomplete suppression of lobster DNA. Despite use of our blocking primer, 44.7% of ASVs inferred from our metabarcoding samples were still identified as lobster. Additionally, many of the ASVs identified after removal of those ASVs were identified simply as malacostracan, which may still represent lobster DNA; therefore the actual amount of lobster ASVs remaining after manual removal was likely underestimated. Regardless of these difficulties, the number of ASVs identified down to specific taxonomic levels far outstripped the microscopy data, and each additional metabarcoding sample revealed a greater diversity of prey items than microscopy (Fig 5). There were also differences in the prey identified. Microscopy identified arthropods more often, while Protista, Mollusca, Annelida, Craniata (likely fish eggs), Cnidaria, etc. were far more common in metabarcoding samples (Fig 6). In fact, Mollusca and Craniata were entirely undetected through microscopy. Each technique offers slightly different views of the diet. Due to opposing strengths and weaknesses of microscopy vs. metabarcoding, the most accurate portrayal of the diet may require integrating both. Whereas the majority of microscopy data were identified at the class level, 44.5% of ASVs were identified down to the species level; demonstrating the capability of metabarcoding to produce gut content data at taxonomies more specific than microscopy. However, most ASVs had very low RRAs (Fig 4). A low RRA could represent environmental contamination, secondary-consumption, or less common diet items [42,43]. While environmental contamination is to be guarded against, rare diet items may still be consequential, so researchers must examine ASV and RRA data critically with respect to the studied predator’s ecology. Microscopy can help to further determine whether ASVs with low RRAs are true prey items. Additionally, the ASVs identified as putative prey taxa can provide new targets for the highly-focused rtPCR approach.

### rtPCR

Species-specific real-time PCR (rtPCR) assays are a viable alternative to the development of a blocking primer if the aim is to target a particular prey taxon. The *Calanus finmarchicus* rtPCR assay was capable of amplifying a DNA target from postlarval lobster gut samples fed a known diet of *C. finmarchicus* (Fig 7), as well as field-caught postlarval lobster gut samples; providing evidence that postlarvae in the field feed on *C. finmarchicus* (Table 3). Furthermore, one of the field-collected postlarval samples that amplified with the *C. finmarchicus* rtPCR was the same source of DNA as sample 1 from the metabarcoding study. Using the species-specific rtPCR approach, *C. finmarchicus* was detected in this postlarva, even though it was not detected using the universal metabarcoding approach. In fact, *C. finmarchicus* was only detected in 1 out of 28 metabarcoding samples (3.6%) compared to 10 out of 48 in the rtPCR samples (20.8%). This demonstrates the importance of utilizing species-specific primers when specific species are of interest, and illustrates the greater sensitivity of rtPCR compared to metabarcoding for detecting relatively rare DNA targets.

A review of *C. finmarchicus* sequences found in GenBank revealed many sequences that are indistinct from the closely related congener *Calanus glacialis*. There are a few potential causes for two distinct species generating indistinct DNA sequences. One possibility is that the two species are simply very similar at the chosen genetic marker, cytochrome oxidase subunit I (COI). Choice of genetic marker can be influential for a number of reasons to metabarcoding and rtPCR assays alike, including variation in coverage and resolution for different taxa at selected marker sites [44]. It has also been theorized that *C. finmarchicus* and *C. glacialis* may hybridize in their natural environment, which could cause difficulties in distinguishing the two species genetically [45]. Finally, it has also been noted that the two species are so similar in morphology that genetic testing may be the only reliable way of distinguishing the two species [46]. Although we are confident that our *C. finmarchicus* rtPCR assay detects *C. finmarchisus* DNA, we recognize that some (potentially misidentified) sequences from specimens reported in GenBank as *Calanus glacialis* would be detected by our species-specific approach.

Though the rtPCR assay was designed to amplify *Calanus finmarchicus*, there remains the possibility that *Calanus glacialis* hybridized with a *C. finmarchicus* mother would also amplify given the maternal inheritance of the mitochondrial genes including COI. Importantly, our novel rtPCR assay successfully detected the presence of *Calanus sp.* in field-caught postlarval lobster guts. Ecologically, the two *Calanus* species that could have been detected by the assay fulfill similar roles and are each significantly lipid rich, making either one a potentially important prey species to postlarval lobsters. In this case it may even be sensible to use an rtPCR which is species-agnostic for *Calanus*, as the postlarva likely derives the same benefit from the two. However, researchers looking to produce a more specific rtPCR will need to keep in mind that efficacy of the assay may be dependent on marker choice and the ecology, morphology and hybridization potential of their target species.

## Conclusion

As eDNA technology becomes more common and papers referencing eDNA surge [47], there could be a tendency to adopt a “newer is always better” attitude. However, what was made clear in this study is that each technique, from well-established microscopy and contemporary eDNA methods alike, have their own strengths and weaknesses. While individual visual diet analysis by microscopy may be time consuming and biased due to disparate ease in prey identification, this technique is also the cheapest and has the potential to reveal specific information regarding quantity and disposition of prey. This makes microscopy a good candidate for a pilot study prior to or in the beginning stages of a diet study. While microscopy can provide a general idea of the predator’s diet, eDNA techniques can be used to considerably augment the analysis. Metabarcoding studies can have significant benefits in providing a relatively unbiased picture of the diversity of prey taxa in the diet. Still, initial costs can be significant, and necessitate time spent developing a PCR protocol and blocking primer. Additionally, metabarcoding is sensitive to marker choice and completeness of reference libraries, but initial microscopy data could be used to better inform marker choice for targeted prey species. Nonetheless, once a protocol is developed and optimized, metabarcoding can be used to quickly generate large amounts of reproducible diet data less subject to individual researcher biases. Still, because of the considerable power of metabarcoding to resolve even the smallest and least abundant taxa, it is important the interpretation of resulting data be informed by a knowledge of the feeding ecology of the consumer. Utilizing microscopy and metabarcoding together has the added benefit of allowing the researcher to cross-reference and validate each data set; a diet constituent which appears in both is more likely to represent a true prey item, rather than environmental contamination or secondary-predation. Finally, rtPCR, unlike the other two methodologies discussed, can be particularly valuable in identifying species of interest. A species-specific rtPCR assay is the best way to ascertain how frequently a prey taxon is ingested. It is the recommendation of this study that a combination of both microscopy and eDNA techniques are likely to produce the most accurate picture of a planktonic predator’s diet.

## Supporting information

S1 Fig Metabarcoding sampling flowchart. This flowchart depicts how postlarval guts were allocated to either grouped, single, or pre-amplified samples.
(JPG)

S1 File Data. This excel file contains the data used in this study.
(XLSX)
