# Supplementary figures and images for: Contemporary eDNA methods complement conventional microscopy in zooplankton diet studies: Case study with American lobster postlarvae
Source: PLoS One. 2025 Jun 25;20(6):e0325889. doi: 10.1371/journal.pone.0325889 (PMC12194154; doi:10.1371/journal.pone.0325889)

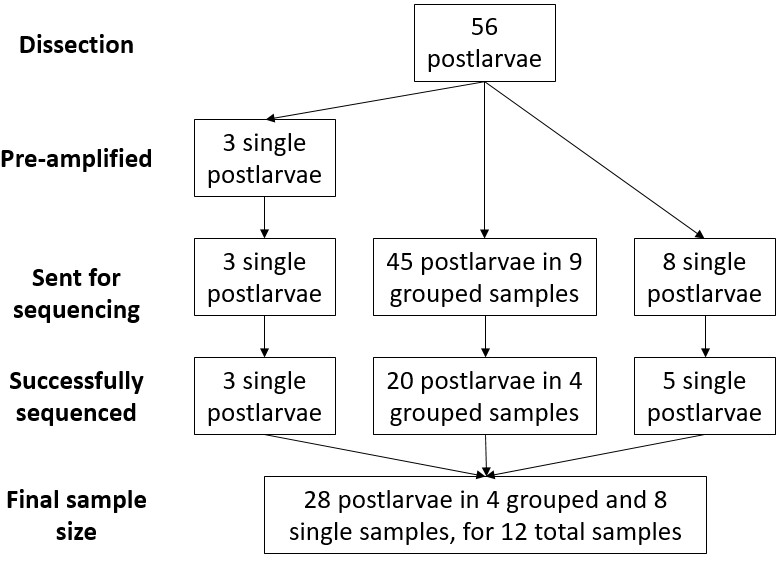

Supplement: S1 Fig — This flowchart depicts how postlarval guts were allocated to either grouped, single, or pre-amplified samples. (JPG) [file pone.0325889.s001.jpg]
